# Genotoxicity and Cytotoxicity Evaluation of the Neolignan Analogue 2-(4-Nitrophenoxy)-1Phenylethanone and its Protective Effect Against DNA Damage

**DOI:** 10.1371/journal.pone.0142284

**Published:** 2015-11-10

**Authors:** Alex Lucas Hanusch, Guilherme Roberto de Oliveira, Simone Maria Teixeira de Sabóia-Morais, Rafael Cosme Machado, Murilo Machado dos Anjos, Lee Chen Chen

**Affiliations:** 1 Instituto de Ciências Biológicas, Universidade Federal de Goiás, Goiânia, GO, Brazil; 2 Instituto de Química, Universidade Federal de Goiás, Goiânia, GO, Brazil; IIT Research Institute, UNITED STATES

## Abstract

Neolignans are secondary metabolites found in various groups of Angiosperms. They belong to a class of natural compounds with great diversity of chemical structures and pharmacological activities. These compounds are formed by linking two phenylpropanoid units. Several compounds that have ability to prevent genetic damage have been isolated from plants, and can be used to prevent or delay the development of tumor cells. Genetic toxicology evaluation is widely used in risk assessment of new drugs in preclinical screening tests. In this study, we evaluated the genotoxicity and cytotoxicity of the neolignan analogue 2-(4-nitrophenoxy)-1-phenylethanone (4NF) and its protective effect against DNA damage using the mouse bone marrow micronucleus test and the comet assay in mouse peripheral blood. Our results showed that this neolignan analogue had no genotoxic activity and was able to reduce induced damage both in mouse bone marrow and peripheral blood. Although the neolignan analogue 4NF was cytotoxic, it reduced cyclophosphamide-induced cytotoxicity. In conclusion, it showed no genotoxic action, but exhibited cytotoxic, antigenotoxic, and anticytotoxic activities.

## Introduction

Neolignans are secondary metabolites found in various groups of Angiosperms. They belong to a class of natural compounds with great diversity of chemical structures and pharmacological activities. They are formed by connecting two phenylpropanoid units via a C-C bond different from β-β' [1].

The oxyneolignan group exhibits a great variety of biological properties such as anti-leishmanial, antioxidant, and antitumor activities [2–4]. Among these compounds, 8-*O*-4' type neolignans are known to exhibit antifungal, antioxidant, anti-inflammatory, antitumor, and antinitric oxide production activities [4–7]. Since the neolignan analogue 2-(4-nitrophenoxy)-1-phenylethanone (4NF) is a synthetic 8-*O*-4' type oxyneolignan, the investigation of its biological activities can provide information to help develop new drugs [8].

Many drugs present genotoxic risks and can cause changes in the genetic material of germ and/or somatic cells, accelerating carcinogenesis and the cell aging process [9]. In order to evaluate the genotoxic and carcinogenic risks for humans, the regulatory authorities of the European Union, Japan, and the United States recommend that genotoxic and carcinogenic evaluations are conducted before application for marketing approval of pharmaceuticals [10].

Important factors for lengthening human life span are cancer prevention and tumor control. It has been shown that many phytochemicals present potential to prevent DNA damage. The search for plants and phytochemicals that present antigenotoxic and cytotoxic activities is of great importance for the development of chemopreventive agents to be used in cancer therapies [11–14].

Several tests can be performed to assess the genotoxicity and cytotoxicity of phytochemicals. The mouse bone marrow micronucleus test has been extensively used in genotoxicity studies of chemicals. It is a simple and quick technique to evaluate the genotoxicity and cytotoxicity of compounds, originally developed using bone marrow cells of mice, but alternatively it can also use the erythrocytes of peripheral blood [15].

Another important test for genotoxicity evaluation is the comet assay or single cell gel electrophoresis (SCGE), which is a rapid and sensitive method to measure and detect double-strand breaks, single-strand breaks, incomplete excision repair sites, and alkali-labile sites in individualized eukaryotic cells. It is suitable to detect clastogenic compounds, but not aneugenic compounds. This test is used to evaluate the genotoxic effect of physical, chemical, or biological agents. It is based on the use of individual cells that have their membranes lysed and most of the proteins removed, allowing the movement of DNA during the electrophoretic run conducted in an alkaline buffer [16–18].

The aim of this study was to evaluate the genotoxic and cytotoxic activities of the neolignan analogue 2-(4-nitrophenoxy)-1-phenylethanone (4NF) in mice and its protective effect against DNA damage.

## Materials and Methods

### Chemicals

4NF was obtained by the reaction between phenacyl bromide and *p*-nitrophenol in basic medium [19]. Under these conditions the product was obtained in 84% yield.

Cyclophosphamide was purchased from Hera Medicamentos (Belo Horizonte, MG, Brazil), fetal bovine serum from Laborclin (Campinas, SP, Brazil), and Giemsa from Doles (Goiânia, GO, Brazil). All the other reagents used were purchased from Vetec Química Fina Ltda (Duque de Caxias, RJ, Brazil) and Synth (Diadema, SP, Brazil).

### Animal testing

This study was approved by the Ethics Committee on Animal Use of the Universidade Federal de Goiás (protocol no. 060/13). The experiments followed national and international standards of management and experimentation with animals [20,21].

Healthy, young, male adult outbred mice (*Mus musculus*, Swiss Webster), between 7 and 12 weeks old, weighing 30–40 g, obtained from the animal facilities of the same university, were randomly allocated to treatment groups. All animals were brought to the laboratory seven days before the experiments and housed in polyethylene cages (40 cm × 30 cm × 16 cm), lined with wood shavings, in groups of five animals, in air-conditioned rooms kept at 25 ± 2°C and 50 ± 10% relative humidity, with a 12-h light/dark natural cycle. Standard food pellets and water were provided *ad libitum*.

#### Treatments

The mice were divided in groups of five animals each, treated intraperitoneally (i.p.) with 4NF at the doses of 50 mg/kg, 75 mg/kg, and 100 mg/kg dissolved in dimethylsulfoxide (DMSO). To evaluate the possible protective effect of the neolignan analogue against cyclophosphamide-induced damage, mice simultaneously received cyclophosphamide at the dose of 50 mg/kg i.p. The experiments were conducted for 24 h and 48 h. A positive control group (50 mg/kg cyclophosphamide i.p.) and two negative groups (10 μL/g DMSO and 10 μL/g saline) were also included.

#### Mouse bone marrow micronucleus test

Experiments were performed according to von Ledebur and Schmid [22]. The animals were euthanized by cervical dislocation under sodium pentobarbital anesthesia and their bone marrow cells were flushed from both femurs in fetal calf serum. After centrifuging (300xg, 5 min), the bone marrow cells were smeared on glass slides. The slides were fixed in absolute methanol for 10 min and stained with buffered Giemsa solution at pH 6.8 for 10 min [15].

Slides were analyzed under light microscope (1000x) to detect possible changes and/or chromosomal losses in young red blood cells of bone marrow of mice submitted to the different treatments. The number of micronucleated polychromatic erythrocytes (MNPCE) in 2000 polychromatic erythrocytes (PCE) was assessed using two slides per animal, whereas cytotoxicity and anticytotoxicity were evaluated by calculating the ratio of PCE to normochromatic erythrocytes (NCE) [23].

#### Comet assay

The comet assay was carried out under alkaline conditions according to the standard procedures described by Singh [24]. The same mice treated with 4NF in the micronucleus test were used in this assay.

Slides previously coated with normal melting point agarose (1.5%) received a homogenate of peripheral blood cells with low melting point agarose 0.75% at 37°C. The material was spread on the slides with coverslips and taken to a cold chamber. After gelation, the coverslips were carefully removed. The slides were immersed in lysis solution (1% triton X-100, 10% DMSO, 2.5 M NaCl, 100 mM Na_2_EDTA, and 10 mM Tris, pH 10.0) for 12–24 h, at 4°C. Subsequently, the slides were incubated with freshly made alkaline solution (300 mM NaOH, 1 mM EDTA, pH > 13) for 20 min, at 4°C for DNA unwinding. The electrophoresis was performed in the same buffer at 300 mA and 1 V/cm for 25 min at 4°C. After the electrophoresis, the slides were placed in a staining tray, covered with a neutralizing buffer (0.4 M Tris-HCl, pH 7.5). The slides were then stained with ethidium bromide and analyzed.

Nucleoid images were captured with an epifluorescence Leica DM 2000 Citogen microscope (Leica Microsystems, Wetzlar, Germany), equipped with a Jenoptik ProgRes^®^ MF camera (Optronics, Goleta, CA, USA), driven by Lucia Cytogenetics^TM^ version 2.5 software (Laboratory Imaging Ltd, Prague, Czech Republic). Cell images were analyzed using the Comet Assay Software Project (CASP) version 1.2.3beta2 [24–26]. The calibration parameters used were: head center threshold = 0.95, thresholds comet = 0:05, head threshold = 0:05, tail threshold = 0.1, and profile 1. DNA damage was evaluated assessing 100 nucleoids per animal using the following parameters: percentage of DNA in the tail, tail moment, and Olive tail moment.

### Statistical analysis

The experiments were performed in triplicate, and the values obtained are expressed as mean ± standard deviation. The GraphPad Prism software 5 was used for the statistical analyses. Prior to the hypothesis test, tests of normality and homoscedasticity were applied to data. Homoscedastic data were analyzed using one-way analysis of variance (ANOVA) followed by Dunnett’s post-hoc multiple-comparison test. Heteroscedastic data were analyzed using Kruskal-Wallis test followed by post-hoc Dunn's test. The results were considered significantly different when p < 0.05.

## Results

### Mouse bone marrow micronucleus test

The results of the bone marrow micronucleus test in mice treated with 4NF did not show significant increase in MNPCE frequency at 24 h (p > 0.05) and 48 h (p > 0.05) of exposure (Table 1). Cytotoxicity was calculated by the ratio of PCE/NCE. Only the dose of 100 mg/kg was significantly different from the negative control group at 24 h of exposure (p < 0.05), demonstrating the cytotoxic action of the neolignan analogue 4NF at this dose.

**Table 1:** Mean values of MNPCE frequency and PCE/NCE ratio after treatment with different doses of the neolignan analogue 2-(4-nitrophenoxy)-1phenylethanone (4NF) in mice for genotoxic and cytotoxic evaluation.

Treatment (mg/kg)	24 h		48 h	
	MNPCE/2000PCE	PCE/NCE	MNPCE/2000PCE	PCE/NCE
**Saline (nc)**	4.60 ± 1.82	0.97 ± 0.15	4.60 ± 1.82	0.97 ± 0.15
**DMSO (nc)**	4.40 ± 1.14	1.03 ± 0.20	4.60 ± 1.31	0.98 ± 0.12
**CP (pc)**	36.0 ± 2.60**	0.48 ± 0.07**	26.0 ± 1.60*	0.59 ± 0.17*
**50 mg/kg**	9.20 ± 2.77	0.83 ± 0.27	4.40 ± 1.14	0.93 ± 0.23
**75 mg/kg**	7.60 ± 1.67	0.77 ± 0.12	4.60 ± 1.52	0.78 ± 0.32
**100 mg/kg**	7.00 ± 1.58	0.63 ± 0.18*	4.80 ± 1.67	0.72 ± 0.25

Values expressed as mean ± standard deviation. DMSO, dimethylsulfoxide; CP, cyclophosphamide (50 mg/kg), nc, negative control; pc, positive control.

* p < 0.05

** p < 0.01, compared to the negative control.

The evaluation of antigenotoxicity using the micronucleus test revealed that 4NF at the doses of 75 mg/kg and 100 mg/kg reduced micronucleus frequency induced by cyclophosphamide at 24 h (p < 0.05). At the dose of 100 mg/kg, this neolignan analogue also decreased MNPCE frequency by 52% at 48 h (p < 0.05) (Table 2). In the evaluation of anticytotoxicity the ratio of PCE/NCE increased at 24 h at the dose of 100 mg/kg (p < 0.05) and at 48 h at the doses of 50 mg/kg and 100 mg/kg (p < 0.05), demonstrating a protective action against the cytotoxicity induced by cyclophosphamide.

**Table 2:** Mean values of MNPCE frequency and PCE/NCE ratio after simultaneous treatment with cyclophosphamide and the neolignan analogue 2-(4-nitrophenoxy)-1phenylethanone (4NF) in mice for antigenotoxic and anticytotoxic evaluation.

Treatment (mg/kg)	24 h		48 h	
	MNPCE/2000PCE	PCE/NCE	MNPCE/2000PCE	PCE/NCE
Saline (nc)	4.60 ± 1.82***	0.97 ± 0.15**	4.60 ± 1.82**	0.97 ± 0.15*
DMSO (nc)	4.40 ± 1.14***	1.03 ± 0.20**	4.60 ± 1.31**	0.98 ± 0.12*
CP (pc)	36.0 ± 2.60	0.48 ± 0.07	26.0 ± 1.60	0.59 ± 0.17
CP + 50 mg/kg	34.0 ± 2.60	0.58 ± 0.10	21.0 ± 2.30	0.73 ± 0.07*
CP + 75 mg/kg	20.6 ± 2.10**	0.59 ± 0.20	18.0 ± 1.30	0.67 ± 0.02
CP + 100 mg/kg	17.0 ± 2.50**	0.69 ± 0.17*	12.0 ± 2.90*	0.74 ± 0.11*

Values expressed as mean ± standard deviation. DMSO, dimethylsulfoxide; CP, cyclophosphamide (50 mg/kg), nc, negative control; pc, positive control.

* p < 0.05

** p < 0.01

*** p < 0.001 compared to the positive control.

### Comet assay

**Fig 1:**
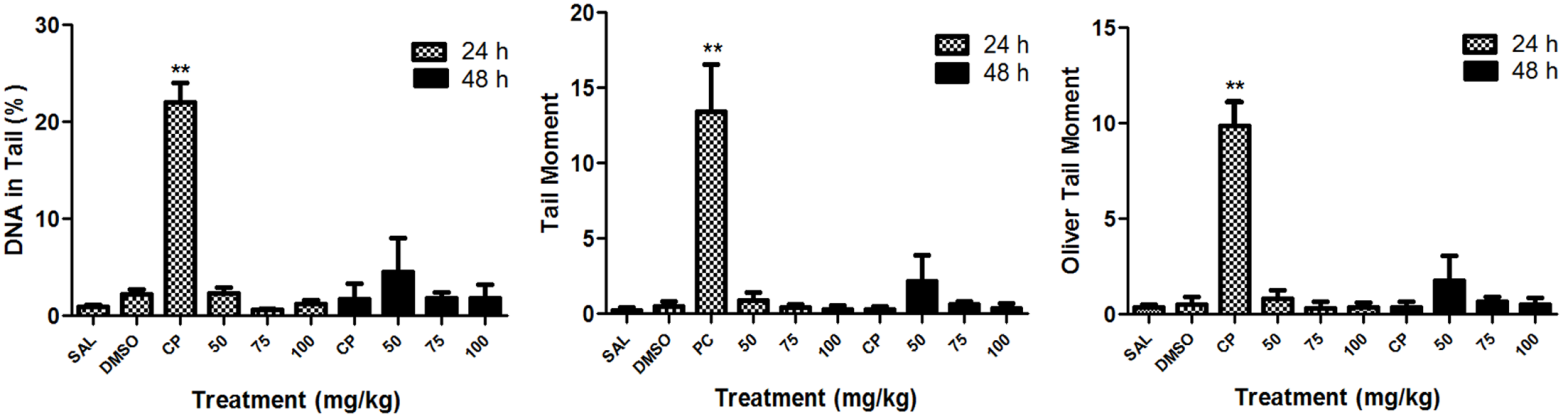
Assessment of the genotoxic activity of the neolignan analogue 2-(4-nitrophenoxy)-1phenylethanone (4NF) in mouse peripheral blood leukocytes using the comet assay estimated by the parameters percentage of DNA in the tail, tail moment, and Olive tail moment. SAL, saline (negative control); DMSO, dimethylsulfoxide (negative control); CP, cyclophosphamide (50 mg/kg) (positive control). * p < 0.05, ** p < 0.01 compared to the negative control.

The comet assay, applied to evaluate the levels of primary DNA damage in peripheral blood leukocytes of mice treated with 4NF, showed no significant difference between the doses tested and the negative control group for the parameters percentage of DNA in the tail, tail moment, and Olive tail moment at 24 h and 48 h (Fig 1). Therefore, this neolignan analogue exhibited no genotoxic effects at all doses tested.

**Fig 2:**
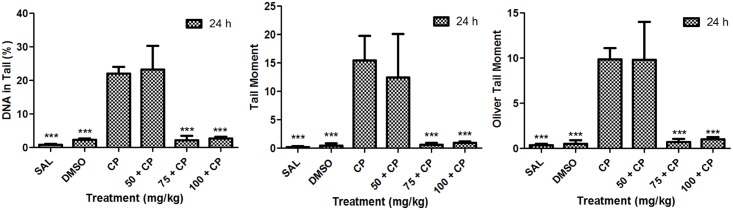
Assessment of the antigenotoxic activity of the neolignan analogue 2-(4-nitrophenoxy)-1phenylethanone (4NF) in mouse peripheral blood leukocytes using the comet assay estimated by the parameters percentage of DNA in the tail, tail moment, and Olive tail moment. SAL, saline (negative control); DMSO, dimethylsulfoxide (negative control); CP, cyclophosphamide (50 mg/kg) (positive control). * p < 0.05, ** p < 0.01, *** p < 0.001 compared to the positive control.

Antigenotoxicity evaluation showed decrease in the values of all parameters assessed using the comet assay at the doses of 75 mg/kg and 100 mg/kg at 24 h of exposure (p < 0.05). However, at 48 h the values were close to zero for all treatments, including the positive control with cyclophosphamide, which limited the measurement for this time of exposure (Fig 2).

## Discussion

Genotoxicity tests are of fundamental importance for drug development, since genotoxic compounds can cause mutation and chromosomal damage events that are essential to initiate carcinogenesis [27]. The pharmacological and toxicological activities of the neolignan analogue 4NF have not been studied yet. In our study, this compound showed no significant genotoxic activity in mice at the doses tested assessed by the micronucleus test in bone marrow as well as by the comet assay in peripheral blood leukocytes.

Studies with neolignans extracted from plants also showed no genotoxic effects induced by these compounds. The possible genotoxic effects of a plant extract containing 94% of magnolol neolignan and 1.5% of honokiol neolignan were investigated. The authors concluded that the standardized extract showed no mutagenic activity using the Ames mutagenicity test and did not induce micronucleus formation in immature erythrocytes of Swiss albino mice [28].

Similar results were obtained studying the lignan daurinol. The compound showed no genotoxic effect on human peripheral blood leukocytes using an *in vitro* test [29]. The lignan cubebin showed no genotoxic action in a study employing the mouse bone marrow micronucleus test [30].

The antitumor activity of neolignans due to the presence of cytotoxic action has also been tested [4]. Cytotoxicity of natural compounds has received great attention during recent years due to their important role in the prevention and treatment of diseases, as well as in the development of promising chemotherapeutic candidates. In the last 20 years, over 60% of the new drugs for the treatment of cancer are of natural origin [4]. The neolignan analogue 4NF showed cytotoxicity due to the decrease in PCE/NCE ratio in the bone marrow of mice at the dose of 100 mg/kg (p < 0.05).

Studies have found lignoids with cytotoxic activity specific for some tumor cell lines, such as neolignan licarin A and the lignans galbacin, sesamin, machilin A and G. These lignoids were effective in inhibiting proliferation of tumor cell lines A549 (human lung carcinoma), MCF-7 (human breast adenocarcinoma), and HCT-15 (human colon adenocarcinoma) by inhibition of phospholipase Cγ1, which is an important factor for tumor cell proliferation. Lignan sesamin proved to be cytotoxic to tumor cell lines CCRF-CEM and CEM/ADR5000 (human T-leukemia) [31,32].

Antigenotoxic and antimutagenic compounds have chemopreventive properties against genotoxins. In the present study, the neolignan analogue 4NF was able to significantly reduce cyclophosphamide-induced cytotoxic and genotoxic damages assessed by the mouse bone marrow micronucleus test and the comet assay. The neolignan grandidisin also reduced MNPCE frequency in mice treated with cyclophosphamide, a known genotoxic agent [33].

The neolignan magnolol has shown to be antimutagenic as assessed by the Ames mutagenicity test using *Salmonella typhimurium* strains TA98 and TA100 [34]. The same compound also showed antigenotoxic activity using the mouse bone marrow micronucleus test. Additionally, magnolol inhibited oxidative damage induced by X-ray due to an increase in the activity of catalase, superoxide dismutase, glutationa-transferase, and uridine diphosphate-glucuronosyl transferase, enzymes that act in the detoxification process [35].

The lignin hinokinin is able to reduce the chromosomal damage caused by methyl methane sulfonate (MMS) acting as a desmutagenic substance [36]. Hinokinin showed no genotoxic activity, but proved to be antigenotoxic in an *in vivo* trial with Wistar rats previously treated with doxorubicin [37].

The lignan cubebin was able to inhibit the genotoxicity induced by doxorubicin due to its antioxidant activity [33]. This action has been identified by the determination method using 2,2-diphenyl-1-picrylhydrazyl (DPPH), and this compound was considered a desmutagenic agent [38].

## Conclusion

The neolignan analogue 4NF did not exhibit genotoxic effect at the tested doses. This compound was able to reduce cytotoxic and genotoxic effects of cyclophosphamide. The possible use of the neolignan analogue 4NF as a chemopreventive and/or therapeutic agent still requires further investigation using different mutagenicity/genotoxicity tests, long-term tests in animals, and research in molecular biology to carry out comprehensive studies of gene expression.
